# Transactions of the Medical Association of Alabama

**Published:** 1875-10

**Authors:** 


Transactions of the Medical Association of Alabama. Twenty-Fifth Annual Session. 1875; pp. 356.

This volume, containing 356 pages, is highly entertaining and scientific throughout. Articles from some of the most learned medical men of the country fill its pages.

The address of the President on hygiene, etc., is well prepared, and, if the suggestions made are carried out, much good will result.

The Annual Address, by Dr. Geo. A. Ketchum, of Mobile, on Medical Ethics, was replete with wisdom. This gifted orator closed his remarks with these words:

"Gentlemen of the medical profession—prepare to do honor to the true science of medicine. Learn well its law and principles, obey the mandates of its ethics, and wage eternal warfare on all systems of quackery that seem to undermine its secure foundations. What, though quackery and empiricism flourish for the hour! Why heed the puny efforts of Homœopathy and its kindred delusions to divert the stream of science from its accustomed channels? They are but dreams and shadows—visions springing from human credulity and human curiosity. Where are their temples and their monuments? Where their literature—their contributions to science? Where their commissions—where the demands made upon them to elucidate the high problems of life and its dependencies—where their multiplied claims upon humanity? No answer comes back through the ages of the past—no voice of the present responds. Whilst true science rears its column high above the crawling things that creep about its base, the names carved in its monumental granite stand out as sharply as ever across the centuries, defying all the puny efforts of sciolists, of isms, and pathies, and self-styled reformers, to deface them."

Prominent among the papers read were the following: Tuberculosis and Scrofulosis, by E. P. Gaines, M.D., Mobile; Climate of the United States considered with reference to Consumption and Pneumonia, by W. D. Bizzell, M.D., of Mobile; Malaria: Its Nature and Mode of Spread, by Benj. H. Riggs, M.D., Selma;

Recent Progress in Gynecology, by F. M. Peterson, M.D., Greenesboro ; History of the Small-pox Epidemic in the city of Mobile, 1874-'75 ; also, Diphtheria, by Jerome Cochran, M.D., Mobile.

Other interesting papers are found, a synopsis of which was given in a former number of the JOURNAL. Upon the whole, the transactions are valuable, and one of which the profession of Alabama may well feel proud.

